# Removal of fermentation inhibitors from pre-hydrolysis liquor using polystyrene divinylbenzene resin

**DOI:** 10.1186/s13068-020-01828-3

**Published:** 2020-11-12

**Authors:** Caoxing Huang, Yayue Zheng, Wenqian Lin, Yuxuan Shi, Guohong Huang, Qiang Yong

**Affiliations:** 1 Jiangsu Co-Innovation Center of Efficient Processing and Utilization of Forest Resources, College of Chemical Engineering, Nanjing Forestry University, Nanjing, 210037 China; 2 Department of Material and Structural Engineering, Nanjing Hydraulic Research Institute, Nanjing, 210037 China

**Keywords:** Pre-hydrolysis liquor, Polystyrene divinylbenzene resin, Fermentation inhibitors, Fermentability, Bioconversion

## Abstract

**Background:**

The presence of soluble lignin, furfural and hydroxymethylfurfural (HMF) in industrial pre-hydrolysis liquor (PHL) from the pulping process can inhibit its bioconversion into bioethanol and other biochemicals. Although various technologies have been developed to remove these inhibitors, certain amounts of sugars are also inevitably removed during the treatment process. Hence, polystyrene divinylbenzene (PS-DVB) resin was used as an adsorptive material to simultaneously remove fermentation inhibitors while retaining sugars with high yields to improve the fermentability of PHL after acid hydrolysis by enriching its xylose concentration. The fermentability of acid-hydrolyzed PHL (A-PHL) was evaluated by the bioconversion into ethanol and xylosic acid (XA) after treatment with PS-DVB resin.

**Results:**

The results showed that the highest xylose concentration (101.1 g/L) in PHL could be obtained by acid hydrolysis at 100 °C for 80 min with 4% acid, while the concentration of fermentation inhibitors (furfural, HMF and lignin) in PHL could also be significantly improved during the acid-hydrolysis process. After treatment with PS-DVB resin, not only were 97% of lignin, 92% of furfural, and 97% of HMF removed from A-PHL, but also 96% of xylose was retained for subsequent fermentation. With resin treatment, the fermentability of A-PHL could be improved by 162–282% for ethanol production from A-PHL containing 30–50 g/L xylose and by 18–828% for XA production from A-PHL containing 90–150 g/L xylose.

**Conclusions:**

These results confirmed that PS-DVB resin can remove inhibitors from PHL before producing value-added products by bioconversion. In addition, this work will ideally provide a concept for producing value-added chemicals from pre-hydrolysis liquor, which is regarded as the waste stream in the pulping process.

## Background

Pre-hydrolysis is a necessary first step in kraft-based dissolving pulp production at pulp and/or paper mills [1, 2]. Steam or hot water is used as medium to treat wood chips during the pre-hydrolysis process, which acts to hydrolyze the majority of hemicellulose and a portion of lignin from the cell wall into a prehydrolysis liquor (PHL) stream. Carbohydrates, furfurals and lignin fragments in PHL exist in the forms of solutes, particulates, and colloids, and PHL is typically regarded as a waste stream [3, 4]. Currently, this liquor is just regarded as the wastewater, which is mixed with the black liquor and introduced into the recovery boiler for energy generation [5]. However, the dissolved hemicellulose in PHL only possesses a heat value of 7400 BTU/b, which is significantly lower than that of the lignins that dominate black liquor (11,300 BTU/b for softwood, 10,600 BTU/b for hardwood) [6, 7]. This means that the current strategy for disposing of PHL actually reduces the energy efficiency of kraft pulping operations. Because the produced PHL contains various amounts of monomeric and oligomeric sugars, as well as soluble lignin, it is possible to envision the use of this waste stream to generate high value. This strategy is in line with the overall biorefinery concept, where multiple value-added products are generated from lignocellulosic materials by different processes [8–10].

Xylose is an important industrial commodity chemical that can be used to produce xylitol, a valuable chemical to both the pharmaceutical and food industries [11]. In the biorefinery concept, xylose can be sustainably used to produce bioethanol by fermentation, which is currently obtained from starch-based crops. Developing bioethanol has been regarded as one of the approaches towards reducing CO_2_ emissions from fossil fuel consumption [12]. Recently, xylonic acid (XA), one of the top 30 high-value chemicals in National Renewable Energy Laboratory (NREL) and Pacific Northwest National Laboratory (PNNL) reports, can also be produced from xylose by chemical, electrochemical, or fermentative methods [13, 14]. Due to the potential applications and great demand for xylose, various types of biomass have been explored at different scales for producing xylose via an acid hydrolysis process [15, 16]. As can be expected, the technologies used for acid hydrolysis processes require carefully orchestrated plans for funding and maintenance during construction, operation, and management [17]. Considering the current industrial presence of xylose in PHL, efforts should also be carried out to upgrade the stream into value-added sustainable chemicals (as opposed to burning it). Such an approach will open an opportunity for producing biochemicals from wastewater in most existing pulp mills that conduct pre-hydrolysis processes.

During prehydrolysis, a small portion of lignin can be depolymerized into water-soluble oligomers. Jiang et al. [18] found that the extensive lignin degradation occurs during prehydrolysis by homolytic cleavage of aryl-ether bonds, resulting in PHL containing an abundance of aromatic compounds in the form of vanillin and syringaldehyde. Furfural and hydroxymethylfurfural (HMF) also exist in PHL due to monosaccharide dehydration during prehydrolysis [19–21]. All these aforementioned monosaccharide byproducts are regarded as fermentation inhibitors for the microbial production of bioethanol, XA, and other biochemicals [22, 23]. Therefore, to efficiently utilize the xylose present in PHL, these inhibitors should be removed from PHL to improve the fermentability and xylose consumption efficiency.

Various methods have been explored for removing inhibitors from PHL. For example, acidification has been used to treat the PHL, however only 3.8% of the soluble lignin was rendered as a filterable precipitate [24]. Wang et al. [25] used membrane filtration with different molecular mass cutoffs to treat PHL and found that the method had limited purifying ability due to overlapping between molecular weight distributions in the carbohydrate fractions. The addition of polydiallyldimethylammonium chloride (p-DADMAC) and cationic polyacrylamide (CPAM) polymers to PHL has also been carried out to flocculate the lignin and lignin-derived products [25]. Yasarla and Ramarao [26] found that flocculating agents with trivalent cations resulted in a 40% loss of sugar despite achieving 70% lignin removal. An adsorption concept using lime mud and activated carbon has also been employed to remove inhibitors in PHL. However, these approaches still cause considerably undesirable sugar adsorption/loss (~ 15–20%). Each of the aforementioned techniques is unable to simultaneously remove solubilized lignin and lignin-derived phenolic substances without hampering the recovery of sugars due to their similar molecular weight distribution and water solubility in PHL.

Differences in polarity between sugars (hydrophilic) and solubilized lignin (hydrophobic) could be exploited to more selectively fractionate inhibitors from target monosaccharides [18]. An approach for selective adsorption using porous resins comprised materials whose chemical structures favors interaction with fermentation inhibitors can ideally be proposed. Research already exists related to lignin-selective adsorptive resins, specifically polystyrene divinylbenzene (PS-DVB) resin [27]. It has been reported that this resin possesses the ability to effectively adsorb soluble lignin and carbohydrate dehydration products while retaining most of the carbohydrate in biomass hydrolyzates due to its hydrophobic polymeric particles that can selectively adsorb similarly hydrophobic solutes through π–π′ and van der Waal interactions [27, 28]. Different from the aforementioned separation methods, the adsorbed lignin in the resin can be desorbed and potentially processed for further application. In most published works, PS-DVB resin was only used to purify biomass hydrolyzate to obtain lignin for further applying or to obtain purified lignin and lignin–carbohydrate complexes for structural analysis [18, 27, 29, 30]. Few studies have used PS-DVB resin as a purification technology to remove the soluble lignin and carbohydrate dehydration products in PHL to improve its fermentability for ethanol and XA production. Hence, it is speculated that PS-DVB resin could be an ideal adsorptive material to simultaneously separate lignin-derived substances and sugars with great efficiency while still improving the fermentability of PHL.

In the present work, acid treatments under different conditions were carried out to hydrolyze PHL xylooligosaccharides into xylose to maximize the quantity of available fermentation substrate. Next, a PS-DVB resin adsorption protocol with different feed flow rates was applied to remove soluble lignin, furfural and HMF. The efficiency and selectivity of this process were verified by compositional analysis of each stream and nuclear magnetic resonance (NMR) characterization. Furthermore, original A-PHL and resin-treated A-PHL with different concentrations were used to produce ethanol and XA in order to evaluate the effects of PS-DVB adsorption on the fermentability of A-PHL. This work will ideally provide a concept to produce value-added chemicals from pre-hydrolysis liquor, which is regarded as the waste stream in the pulping process.

## Results and discussion

### Composition analysis of the PHL

Pre-hydrolysis is the primary technology used for kraft-based dissolving pulp production. During the pre-hydrolysis stage, most hemicellulose and portion of lignin can be removed, which are depended upon both treatments conditions for different woody feedstocks [19, 25]. In consideration of these variables, it is necessary to obtain a detailed chemical composition of the mixed hardwood PHL used in this work. The chemical compositions of PHL are shown in Table 1. It can be seen that xylose (86.3 g/L) and xylooligosaccharide (32.3 g/L) are the major sugars in this PHL. In addition, a certain amount of glucose (7.5 g/L), arabinose (6.1 g/L) and sugar-derived byproducts (0.9 g/L of furfural and 0.4 g/L of HMF) were quantified. The high concentration of xylooligosaccharides in PHL confirms that this stream can be further transformed into xylose-rich solution by hydrolyzing xylooligosaccharides into xylose. The sugar contents in this PHL were significantly higher than those in the PHL (40–60 g/L) used in the work of Shi et al. [3]. This difference occurred because the used mixed hardwood PHL was obtained after steam concentration. In addition, it is important to note that a high concentration (42.1 g/L) of soluble lignin also existed in the PHL.

**Table 1:** Composition analysis of PHL, A-PHL, and P-A-PHL (g/L)

	PHL^a^	A-PHL^b^	P-A-PHL^c^
Xylose	86.3 ± 0.3	101.1 ± 1.1	97.1 ± 0.9
Glucose	7.5 ± 1.2	8.8 ± 0.3	8.4 ± 0.1
Arabinose	6.1 ± 0.2	4.5 ± 0.2	4.3 ± 0.5
HMF	0.4 ± 0.2	1.9 ± 0.1	0.04 ± 0.01
Furfural	0.9 ± 0.4	7.8 ± 1.2	0.6 ± 0.1
Xylooligosaccharide	32.3 ± 2.1	–	–
Glucooligosaccharides	3.3 ± 1.1	–	–
Soluble lignin	42.1 ± 0.5	42.9 ± 0.6	2.1 ± 0.3

^a^Pre-hydrolysis liquor

^b^PHL after acid hydrolysis

^c^A-PHL after purification by PS-DVB resin

**Fig. 1.**
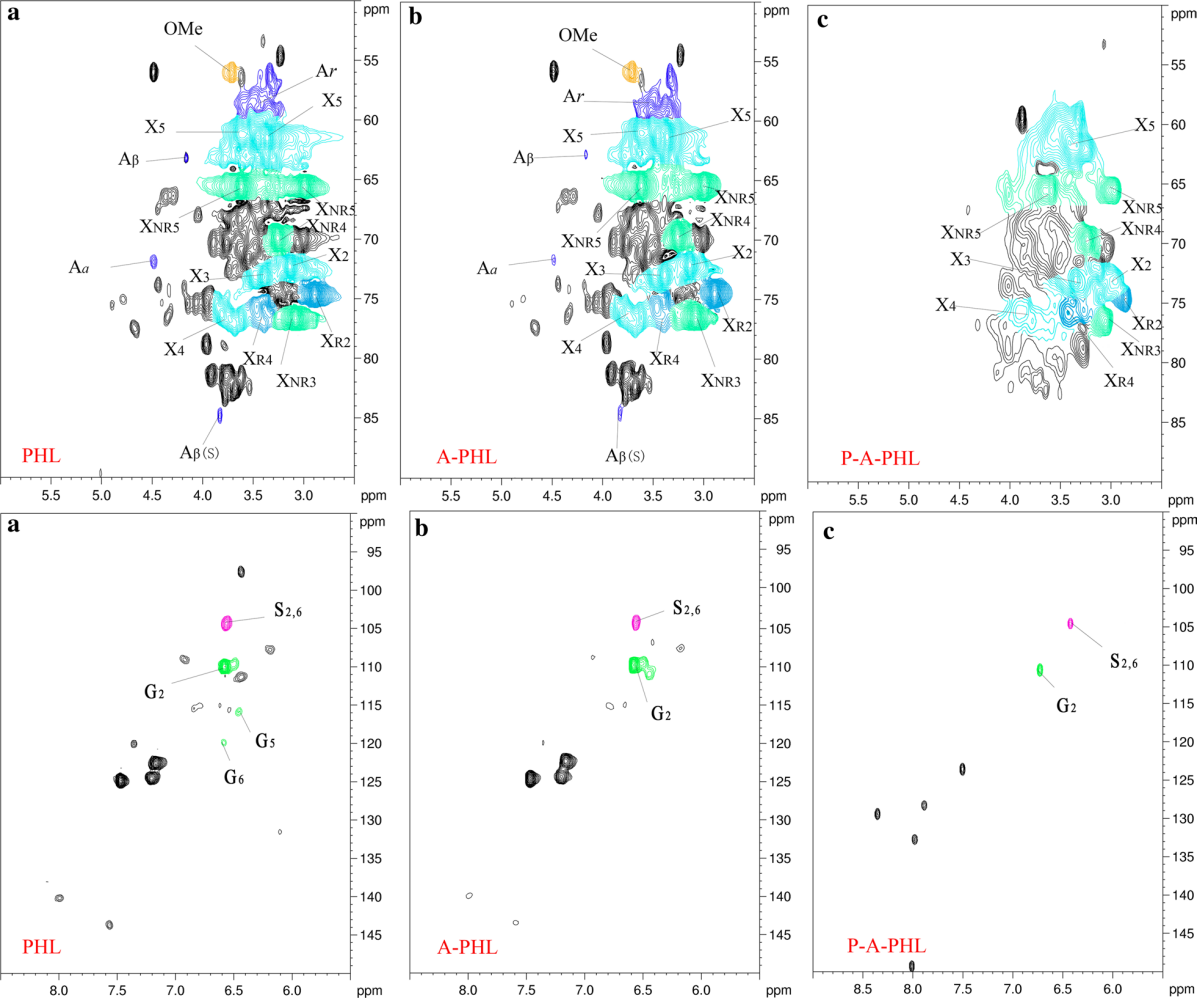
2D-HSQC NMR spectra of PHL (**a**), A-PHL (**b**), and P-A-PHL (**c**)

Two-dimensional heteronuclear single-quantum coherence (2D-HSQC) NMR, an advanced technology that has been used to characterize samples with carbohydrate and lignin mixtures [31] or lignin–carbohydrate complexes [32], was carried out to understand the structural information of the chemical components in the PHL. The obtained spectra are shown in Fig. 1a. Peaks in the 2D-HSQC spectra are assigned according to recent works [33, 34].

The signals for internal xylan units (X) were clear and obvious in the spectra. Specifically, the C_2_/H_2_, C_3_/H_3_, C_4_/H_4_, and C_5_/H_5_ positions showed correlation signals at 72.3/3.03, 73.9/3.22, 75.3/3.48, and 62.9/3.15, 3.85 ppm, respectively. In addition, xylan with reducing-end units (X_R_) was also identified from the C_2_/H_2_ and C_4_/H_4_ correlation signals at 74.4/2.89 and 75.3/3.48 ppm, respectively. The presence of X_R_ indicates that the soluble xylan (xylooligosaccharide) in the PHL possesses a relatively low degree of polymerization, which makes it a promising resource for xylose production [31].

In addition, the spectra of PHL showed common lignin substructures of β-O-4 (A), β-β (B), and β-5 (C), which can be identified by their C_*a*_-H_*a*_ signals at 71.8/4.86, 84.9/4.69, and 86.8/5.49 ppm, respectively. Correlation signals for syringyl units (S) and guaiacyl units (G) were also noted from their C_2_-H_2_ and C_2,6_-H_2,6_ positions at 111.0/7.01 and 104.1/6.74 ppm, respectively. Hence, it can verify the lignin fraction was present in the PHL, which is in accordance with the results of the composition analysis.

### Acid hydrolysis of the PHL

In previous work, it was reported that even though PS-DVB resin shows a low affinity for carbohydrates, some of high molecular weight carbohydrate linked to lignin can still be adsorbed by the resin [27, 29, 30]. To reduce the loss of sugars in PHL and maximize the quantity of available fermentation substrate, the oligosaccharides in PHL were hydrolyzed into monosaccharides. Acid hydrolysis was carried out with sulfuric acid under different reaction conditions (acid concentration, time, and temperature) to obtain a recipe for maximizing xylooligosaccharide conversion into xylose.

**Fig. 2:**
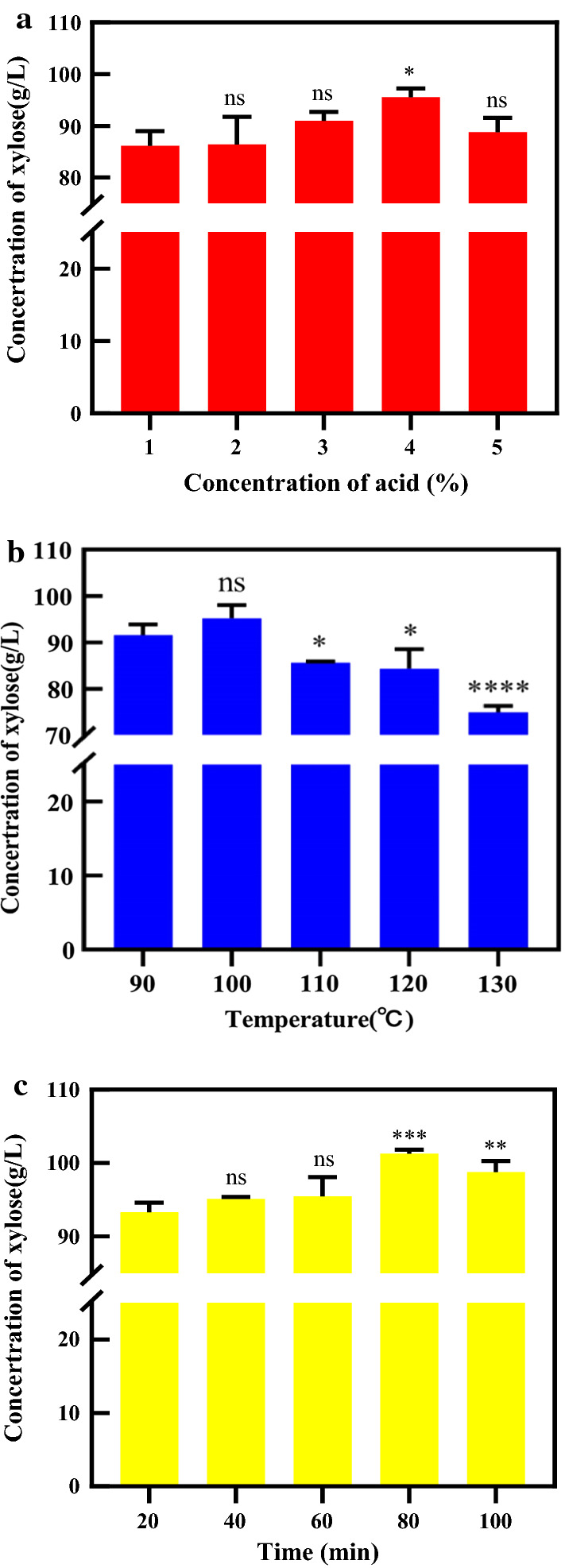
Effects of the acid concentration (**a**), temperature (**b**), and reaction time (**c**) of acid hydrolysis on xylose production [(**a**: temperature: 100 °C; time: 60 min), (**b**: acid concentration: 4%; time: 60 min), (**c**: acid concentration: 4%; temperature: 100 °C)]; ns denotes no significant difference, * denotes *p* < 0.05, ** denotes *p* < 0.01 and *** denotes *p* < 0.001, **** denotes *p* < 0.0001

The increase in xylose concentration in the PHL under different acid concentrations (1–5%) at 100 °C for 60 min is shown in Fig. 2a. First, increasing xylose concentrations were found with increasing acid catalyst concentrations. For example, the concentration of xylose in PHL increased from 86.1 g/mL to 95.4 g/L when the acid concentration was increased from 1 to 4%. However, a maximum was noted, as the xylose concentration decreased to 88.9 g/L when the acid concentration was further increased to 5%. This can be explained by the mechanism that xylose is easily dehydrated into furfural at higher acid concentration. As observed by Yang et al. [35], increasing the acid catalyst (formic acid) concentration from 5 to 10 g/L could effectively dehydrate xylose into furfural, with the yield rising from 15.8 to 74%, indicating that xylose can be degraded with increasing acid concentration. Depending on the maximum xylose concentration in A-PHL, it can be speculated that 4% sulfuric acid was the optimum condition to produce the xylose from PHL.

According to the aforementioned results, the effects of different reaction temperature (90–130 °C) with 4% acid and 60 min of acid hydrolysis on increases in xylose concentration in PHL were investigated and are shown in Fig. 2b. The maximum concentration of xylose in A-PHL was 95.4 g/L at 100 °C. Increasing the reaction temperature from 100 to 130 °C significantly reduced the xylose concentrations from 95.4 to 75.1 g/L. This result occurred because increasing the reaction temperature further accelerated the dehydration of xylose into furfural given the high acid dosage applied in this series of experiments [35]. Chen et al. [31] also found that the xylose in PHL could be further degraded under reaction temperatures from 120 to 135 °C with 4% aqueous sulfuric acid for 30 min. Based on the maximum xylose concentration in A-PHL, it can be speculated that acid hydrolysis with a 4% acid concentration at 100 °C was the optimum condition to produce the xylose from PHL.

The effect of the reaction time (20–100 min) of acid hydrolysis with 4% acid at 100 °C on xylose concentration in PHL is illustrated in Fig. 2c. It can be observed that the xylose concentration in the A-PHL increased first and then decreased with increasing reaction time from 20 to 100 min. The optimal reaction temperature was 80 min, and a maximum xylose concentration of 101.1 g/L was obtained. Therefore, the highest xylose concentration in A-PHL (with a 17% increase relative to that of the original PHL) was obtained by acid hydrolysis at 100 °C for 80 min with 4% sulfuric acid dosage. These conditions approach to those of the work of Chen et al. [31], who found that an optimal xylose yield (30.10 g/L) from poplar PHL was obtained at 120 °C for 0.5 h with 4% aqueous sulfuric acid.

Although a higher xylose concentration can be obtained in PHL by applying acid hydrolysis, this method has also been shown to unavoidable lead to the dehydration of sugars (xylose and glucose) into fermentation inhibitors (furfural and HMF). As seen in Table 1, the PHL subjected to acid treatment under (A-PHL) the aforementioned optimal condition contained a higher xylose concentration than the original PHL. However, the concentration of furfural and HMF were 7.8 g/L and 1.9 g/L, respectively. These quantities are significantly higher than those in PHL (0.9 g/L and 0.4 g/L). In the reported work of Bellido et al. [36], it was found that the pentose-fermenting yeast *P. stipitis* had poor tolerance towards furfural and HMF, with cell growth almost completely inhibited when the fermentation media contained at least 2 g/L furfural. In addition, various phenolic compounds formed from the degradation of lignin can inhibit the efficiency of ethanol fermentation. Zhou et al. [37] found a significant inhibitory effect of furfural occurred using a *G. oxydans* fermentation system with prehydrolyzate containing 6.5 g/L furfural, revealing the toxicity of furfural for XA production from *G. oxydans.* In addition, the concentration of water-soluble lignin (44.9 g/L) was slightly increased in A-PHL relative to that in PHL. As pointed out by Wang and Chen [38], the high concentration (> 2 g/L) of lignin fractions in the sugar solutions can also cause an inhibitory effect on fermentation. Thus, removing the fermentation inhibitors in A-PHL not only provides a favorable environment for pentose-fermenting yeast but also removes a critical hurdle towards the fermentation of xylose to XA by *G. oxydans*.

### Purification of PHL by PS-DVB resin

**Fig. 3:**
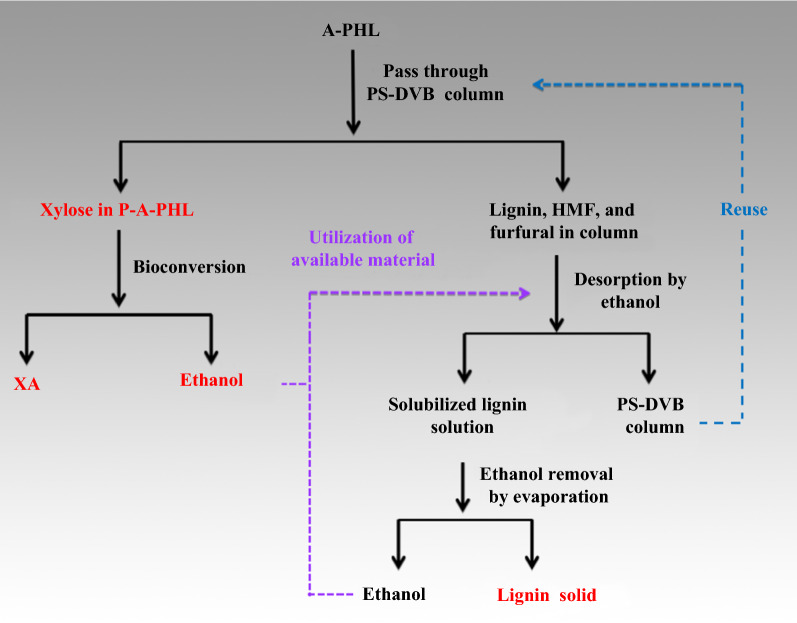
Proposed process diagram for the removal of lignin and sugar byproducts (furfural and HMF) from PHL

Lignin and sugar-derived byproducts in PHL are difficult to separate from sugars due to their similar molecular weights and water solubilities [27]. In this work, we used a lignin-selective adsorptive resin to separate the lignin and sugar byproducts (furfural and HMF) from A-PHL while retaining most of the sugars in the A-PHL. A diagram of the process used is shown in Fig. 3. The lignin and sugar byproducts in A-PHL can be adsorbed in the resin, while the purified sugar solution proceeds to the next unit operation. Ethanol can be used to regenerate the resin by desorbing the adsorbates and can also be recycled to prepare lignin-based materials. Importantly, the regenerated resin can be reused in another purification process to adsorb the lignin and sugar byproducts in A-PHL. Hence, the present concept for the purification of A-PHL by PS-DVB resin is inherently environmentally friendly and sustainable.

First, the most important parameter for resin separation technology, the feed flow rate, was optimized at 0.5–4 mL/min for the column (40 × 2 cm) with a solution loading of 500 mL for adsorption. As shown in Additional file 1: Table S1, the resin’s ability to remove fermentation inhibitors (soluble lignin, furfural and HMF) was similar with an increase in flow rate from 0.5 to 2 mL/min. However, increasing the flow rate from 2 to 4 mL/min resulted in a decreased removal efficiency for fermentation inhibitors, even though there was no obvious decrease in sugars in the treated A-PHL. This result can be attributed to the fact that the higher flow rate might cause the solution (A-PHL) to pass through with a shorter retention time in the resin column, resulting in insufficient time for the PS-DVB resin to adsorb the fermentation inhibitors. This phenomenon is in agreement with the work of Lin et al. [39], who found that a higher resin feed flow rate could result in a higher Reynolds number and prolong the mass transfer zone, leading to a shorter breakthrough time and then decreasing the diffusion coefficient of the resin for the adsorption of substances in aqueous solution. Based on the best fermentation inhibitor removal performance of PS-DVB with the highest retention of xylose for subsequent fermentation, a feed flow rate of 2 mL/min was chosen as the optimal condition for the use of the resin.

As shown in Table 1, the concentration of soluble lignin, HMF, and furfural in PS-DVB treated A-PHL (P-A-PHL) were 1.1 g/L, 0.04 g/L, and 0.61 g/L, respectively. Each of these quantified values is significantly lower than that in the original A-PHL solution (46.9 g/L, 1.9 g/L, and 7.8 g/L, respectively). These results indicate that not only can 97% of the lignin be removed, but also 92–97% of sugar byproducts can be separated from the xylose solution. In addition, a high r proportion (96%) of xylose was retained in P-A-PHL. These results indicate that this process can simultaneously separate and recover lignin and sugars in PHL with recovery > 90%, which is more effective than the reported technologies of acidification, nanofiltration or microfiltration, applying cationic polymers (e.g., p-DADMAC), activated carbon, or lime mud [1, 19, 25]. Overall, the low concentration of fermentation inhibitors in P-A-PHL suggests that the fermentability of A-PHL should be sufficient for producing ethanol and XA, which will be verified in the subsequent work.

To further understand the structural changes of the carbohydrates and lignin in A-PHL after being treated by PS-DVB resin, 2D-HSQC spectra of A-PHL and P-A-PHL were obtained and are shown in Fig. 1b, c, respectively. A-PHL showed the similar cross-signals for various substructures of lignin and carbohydrate in the 2D-HSQC spectra compared to those in the original PHL spectra (Fig. 1a). However, the signal intensities of β-O-4 substructures in the A-PHL spectra were significantly lower than those in the PHL spectra. This difference might be due to the cleavage of ether bond under the acidic hydrothermal conditions of acid hydrolysis [40]. For the 2D-HSQC spectra of P-A-PHL, the signals observed for carbohydrates remained in similar positions compared to those noted in the A-PHL spectra (Fig. 1c). Importantly, the correlations of lignin substructures and units were absent in the P-A-PHL spectra. This result again indicates the successful removal of lignin from A-PHL, which is attributed to the adsorption ability of PS-DVB resin.

### Fermentation of A-PHL and P-A-PHL by *Pichia stipitis* (*P. stipitis*) to produce ethanol

**Fig. 4:**
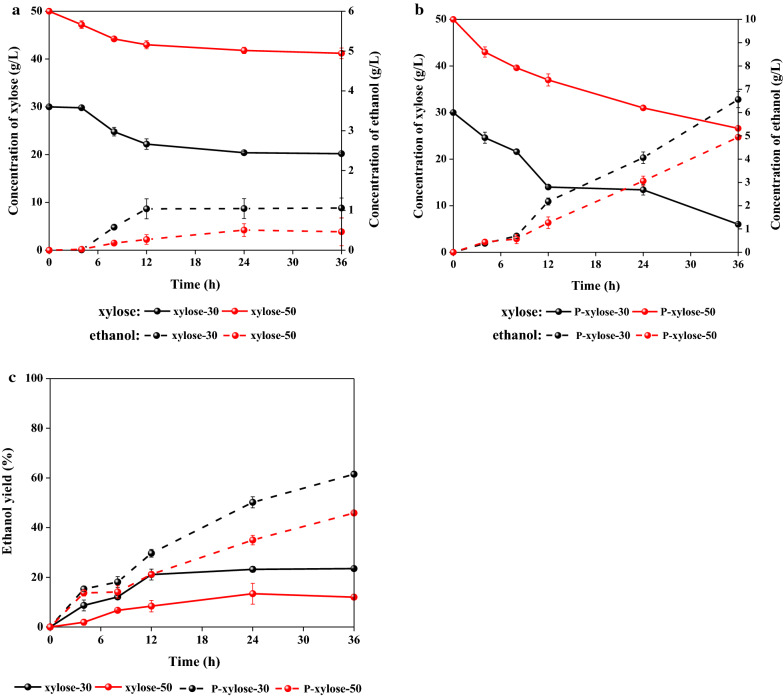
Xylose consumption and ethanol production of A-PHL (**a**) and P-A-PHL (**b**), and their ethanol yields (**c**)

To understand the improvement in the fermentability of A-PHL after being treated by PS-DVB resin, both A-PHL and P-A-PHL (containing the same xylose concentration of 30 g/L and 50 g/L) were used as the fermentation stocks to produce ethanol by *Pichia stipitis* (*P. stipitis*) yeast. The concentration of xylose was selected based on the general tolerance ability of *P. stipitis* for xylose. The fermentation inhibitor concentrations in prepared A-PHL and P-A-PHL containing different xylose concentrations are listed in Table 2. The xylose consumption and ethanol production of A-PHL and P-A-PHL are shown in Fig. 4a,b, respectively. The ethanol yields, which are defined as the percentage of the total amount of ethanol that could be produced from consumed xylose, are shown in Fig. 4c.

**Table 2:** Composition analysis of prepared A-PHL and P-A-PHL containing different xylose concentrations (g/L) for ethanol and xylosic acid fermentation

	A-PHL^a^					P-A-PHL^a^				
Xylose	30.1 ± 0.1	40.2 ± 0.3	50.3 ± 0.1	90.5 ± 0.2	150.6 ± 1.1	30.3 ± 0.3	40.6 ± 0.1	50.3 ± 0.9	90.9 ± 0.3	150 ± 0.1
HMF	0.6 ± 0.5	0.8 ± 0.4	0.8 ± 0.1	1.8 ± 0.4	2.8 ± 0.3	0 ± 0.0	0 ± 0.0	0 ± 0.0	0.1 ± 0.0	0.1 ± 0.0
Furfural	2.4 ± 0.2	3.2 ± 0.3	3.9 ± 0.7	6.7 ± 1.2	10.8 ± 0.9	0.2 ± 0.5	0.3 ± 0.1	0.3 ± 1.1	0.6 ± 0.4	1.2 ± 1.3
Soluble lignin	15.4 ± 1.3	20.1 ± 0.9	23.5 ± 0.8	41.3 ± 0.7	59.9 ± 1.2	0.8 ± 1.1	0.9 ± 0.5	1.0 ± 0.2	2.3 ± 0.4	3.0 ± 1.4

^a^Prepared from corresponding solutions by dilution or concentration

The results in Fig. 4a revealed that A-PHL has low ethanol fermentation productivity with *P. stipitis*. Specifically, xylose in A-PHL was slowly consumed during the fermentation process. After 36 h of fermentation, 20.2 g/L and 41.6 g/L of xylose remained in A-PHL with initial xylose concentrations of 30 g/L and 50 g/L, indicating that only 32.6% and 16.8% of xylose in A-PHL were consumed, respectively. The ethanol yield (Fig. 4c) obtained from A-PHL containing 50 g/L xylose was only 12.0%, which was lower than that of A-PHL containing 30 g/L xylose (23.5%). The low fermentability of A-PHL for producing the ethanol by *P. stipitis* was therefore due to its high concentration of inhibitors [41, 42]. A total of 0.57 g/L HMF and 2.36 g/L furfural were present in A-PHL containing 30 g/L xylose and 0.82 g/L HMF and 3.93 g/L furfural were present in A-PHL containing 50 g/L xylose. As pointed out by Díaz et al. [41], an inhibitory effect on ethanol fermentation by *P. stipitis* can be observed with furfural concentrations of 1–2 g/L, and almost no sugars can be consumed when there is greater than 4 g/L furfural in the fermentation media.

From Fig. 4b, it can be seen that PS-DVB resin treatment remarkably improved the fermentability of A-PHL for ethanol production. 80% and 47% of the xylose in P-A-PHL containing 30 g/L xylose and 50 g/L xylose was consumed after 36 h of fermentation, resulting in ethanol yields of 61.8% and 45.9%, respectively. Compared to the results for untreated A-PHL containing 30 g/L and 50 g/L xylose, the ethanol yield was improved by 162% and 282%, respectively. The improved fermentability of P-A-PHL therefore must be due to the removal of fermentation inhibitors (HMF, furfural, and soluble lignin). As shown in Table 2, the concentrations of HMF, furfural, and soluble lignin in P-A-PHL containing 30 g/L and 50 g/L xylose were 0 g/L, 0.21–0.30 g/L, and 0.75–1.01 g/L, respectively, which were significantly lower than those in untreated A-PHL. In view of the other technologies that have been used to improve the fermentability of sugar solutions with inhibitors, Lai et al. [42] reported that the ethanol production could be increased by 45.5% and 42.8% for the prehydrolyzate treated with cetyltrimethylammonium- and benzyltrimethylammonium-modified bentonites, respectively. Zhu et al. [43] reported that a trialkylamine extraction technology could be used remove 45.7% HMF and 100% furfural from prehydrolyzate, which could improve its fermentability by *P. stipiti*s by a degree of 89.6%. Based on the aforementioned results, it can be concluded that PS-DVB resin treatment is a valid detoxification method to improve A-PHL fermentability for ethanol production.

According to the results in Fig. 4c, the fermentability of A-PHL and P-A-PHL containing 50 g/L xylose was weaker than that of A-PHL and P-A-PHL containing 30 g/L xylose. Two reasons can explain this phenomenon. First, the concentrations of furfural and soluble lignin in A-PHL containing 50 g/L xylose were 3.9 g/L and 0.3 g/L, and those in P-A-PHL containing 50 g/L xylose were 23.5 g/L and 1.0 g/L, respectively; these values were higher than those in A-PHL and P-A-PHL containing 30 g/L xylose. The higher concentrations of these fermentation inhibitors may show greater inhibition of the bioconversion ability of *P. stipitis* to produce ethanol [37]. Second, A-PHL and P-A-PHL containing a higher xylose concentration (50 g/L) may inhibit the bioconversion ability of *P. stipitis.* As pointed out by Agbogbo and Coward-Kelly [44], ethanol productivity can be inhibited when the initial xylose concentration is 50 g/L, which is called sugar inhibition.

### Fermentation of A-PHL and P-A-PHL for producing XA by *Gluconobacter oxydans *(*G. oxydans*)

**Fig. 5:**
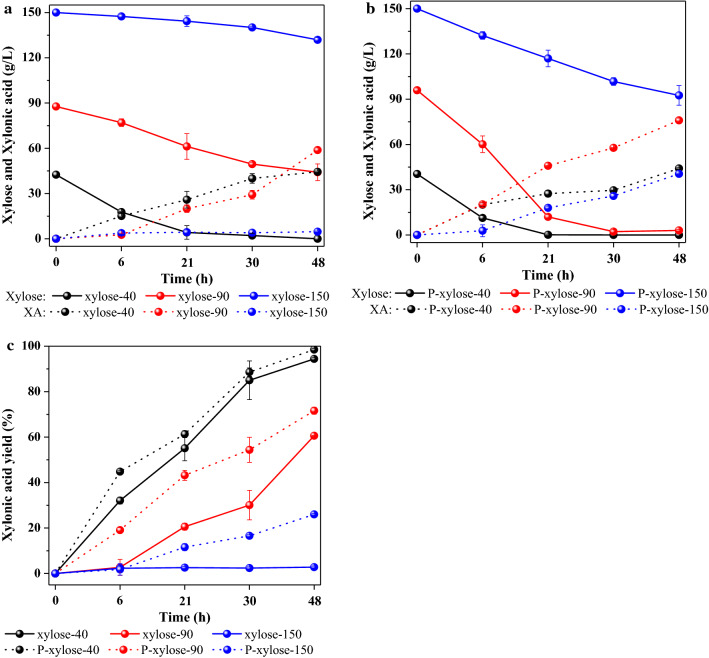
Xylose consumption and XA production of A-PHL (**a**) and P-A-PHL (**b**), and their XA yields (**c**)

The fermentability of A-PHL treated by PS-DVB resin with different xylose concentrations (40, 90, and 150 g/L) to produce XA by *Gluconobacter oxydans* (*G. oxydans*) were investigated. As shown in Fig. 5a, the xylose in A-PHL containing 40 g/L xylose could be completely consumed within 48 h, resulting in an XA production of 44.4 g/L and a corresponding yield of 96.6% (Fig. 5c). When increasing the A-PHL xylose concentration to 90 g/L, even a better XA production of 58.8 g/L could be achieved at 48 h. However, only 49.7% of the xylose was consumed by *G. oxydans* to produce XA, with a yield of 60.6%. When further increasing the A-PHL xylose concentration to 150 g/L, only 12.1% of the xylose could be consumed to produce XA, with a yield of 12.1% (4.7 g/L) at 48 h. From these results, it can be seen that the bioconversion of xylose in A-PHL was strongly inhibited by increases in the concentration of xylose from 40 to 150 g/L, which can be attributed to the corresponding increased concentration of furfural in A-PHL. As can be seen in Table 2, A-PHL containing 40 g/L and 90 g/L xylose contained 3.2 g/L and 6.7 g/L furfural, which were the values within the tolerance of *G. oxydans* to produce XA. However, there was 10.8 g/L of furfural in A-PHL containing 150 g/L xylose, which is beyond the tolerance threshold for bioconversion. As pointed out by Zhou et al. [37], the bioconversion ability of *G. oxydans* can be reduced by 60% when the furfural concentration in fermentation media is increased from ~ 10 to 15 g/L.

Since high concentration of furfural causes severe inhibition of the bioconversion ability of *G. oxydans* [45], we also use the PS-DVB resin to treat A-PHL to produce XA by *G. oxydans*. As seen in Fig. 5b, predictably enhanced XA productivity with 71.6% and 26.1% yields was achieved with P-A-PHL containing 90 g/L and 150 g/L xylose, corresponding to improvements of 18% and 828% compared to the yields of A-PHL containing 90 g/L and 150 g/L xylose, respectively. The tremendous improvement obtained with A-PHL was due to its low concentration of furfural, which was achieved by PS-DVB treatment. According to the research of Chai et al. [46], the inhibition by furfural towards whole-cell catalysis of *G. oxydans* can be avoided by implementation by furfural removal. Our observations support this previous finding. Overall, it can be concluded that PS-DVB resin treatment is also a potential detoxification method for A-PHL to improve its fermentability for XA production, especially when using PHL with high concentrations of xylose and fermentation inhibitors.

### Prospect of the protocol for industry application

In the kraft-based dissolving pulp industry, prehydrolysis is a crucial stage that can result in dissolving the majority of the hemicelluloses and part of the lignin in PHL. Currently, PHL is mostly concentrated with the black liquid from the cooking process and burned in the recovery boiler [4, 7]. Hence, using the generated PHL to produce biobased chemicals means that the cost for the feedstock is almost zero. It is estimated that the global production of dissolving pulp was 5.6 million tons in 2013 and 7.5 million tons in 2015; these quantities can produce 50–80 million tons PHL (nonconcentrated) [47, 48]. The large amount of PHL produced will provide adequate feedstock for the industrial-scale production of ethanol and XA by the current protocol. Acid hydrolysis is a mature and commercial technology for the production of platform chemicals, such as furfural and HMF [49]. In this work, this technology has also been proposed to enrich the xylose amount in PHL to prepare a fermentation substrate. Acid hydrolysis was carried out and optimized at a 4% sulfuric acid dosage at 100 °C for 80 min. These low conditions for preparing the fermentation feedstock indicate that this treatment may result in less reactor corrosion and lower costs for investment, operation, and management [50]. After treatment with PS-DVB, the bioconversion efficiency reached 61.8% for ethanol production from feedstock containing 30 g/L xylose and 96.6% for XA production from feedstock containing 40 g/L xylose. Regarding the final products ethanol and XA, both are important industrial products that can be applied in different areas [11, 13, 14]. Importantly, the used resin can be reused by regenerating it with ethanol, which can be obtained from self-production in the current protocol. Regarding reuse, it can be seen that (Additional file 1: Table S2) after different 5 cycle times, the resin could still remove HFM, furfural and soluble lignin, with reuse efficiencies of 97.9–99.3%, 91.5–90.2%, and 94.2–95.1%, respectively. In addition, the recovery yield of xylose was still over 94% after 5 cycles. The good reuse efficiency of regenerated resin indicated that it has a long lifetime and satisfactory regeneration ability for processing, suggesting potential applications in industry. The adsorbed substances (HFM, furfural and soluble lignin) in the resin can be recovered after being desorbed by ethanol. Due to differences in molecular weight and boiling point, the HFM, furfural and soluble lignin in desorbed ethanol can be further separated by distillation, membrane separation, or other combined technologies. All of these products are important platform chemicals that can be further used to synthesize resins, plastics, and other value-added products [10, 51].

Hence, the potential value-added products produced from A-PHL in the current protocol, such as ethanol, XA, HFM, furfural and lignin, can ideally provide additional revenue streams for dissolving pulp production mills, enhancing their competitiveness [51]. The technology discussed in this work represents a good starting point for future industrial exploitation of PHL due to the low cost of the feedstock, mature technologies, high bioconversion efficiency, satisfactory regeneration of resin, and multiple value-added products. Further research should be carried out to assess industrial applications of this protocol, such as case studies of specific PHLs from different mills, detailed process design, and economic analysis considering costs and revenues.

## Conclusion

Acid hydrolysis was used to treat PHL to enrich its xylose concentration, which was improved from 86.3 to 101.1 g/L with 4% sulfuric acid at 100 °C for 80 min. However, the concentrations of the fermentation inhibitors of HMF and furfural inevitably increased to 1.9 g/L and 7.8 g/L in A-PHL, respectively. Using PS-DVB resin under optimal condition was found to be an effective adsorptive medium capable of simultaneously removing over 90% of fermentation inhibitors while retaining over 95% of monosaccharides. After PS-DVB treatment, the ethanol production yield of A-PHL containing 30 g/L and 50 g/L xylose could be improved by 162% and 282%, respectively. A similarly elevated productivity of 18% and 828% could be achieved for XA production from the same treated A-PHL containing 90 g/L and 150 g/L xylose, respectively. Overall, this work suggests that PS-DVB resin can be regarded as a sustainable technology to improve the bioconversion of prehydrolysis liquor due to its good performance in removing fermentation inhibitors and high reuse efficiency.

## Methods

### Materials

PHL from mixed hardwood, was kindly provided by Sun Paper Co., Ltd (Shandong Province, China). *Pichia stipitis* (*P. stipitis*) yeast and *Gluconobacter oxydans* (*G. oxydans*) were provided by the Biochemical Engineering Research Institute of Nanjing Forestry University. Amberlite^®^ PS-DVB resin (20–60 mesh, 800 m^2^/g) was provided by Dow Chemical Company, USA. Analytical grades of furfural (> 99%), HMF (> 99%), glucose (> 99%), xylose (> 99%), xylosic acid (> 99%), and yeast extract were obtained from Sigma-Aldrich. Sulfuric acid, sodium hydroxide, anhydrous ethanol, MgSO_4_, KH_2_PO_4_, CaCl_2_, K_2_HPO_4_, and (NH_4_)_2_SO_4_ were purchased from Nanhua Chemical Reagent Factory, China. All chemical reagents were purchased from the suppliers and used without purification.

### Acid hydrolysis of PHL

Acid hydrolysis was carried out under different conditions to hydrolyze the xylooligosaccharide into xylose to enrich its amount in PHL. Specifically, a certain amount of concentrated sulfuric acid (98%, w/v) was added to PHL to final acid concentrations of 1–5% (w/v). Acid hydrolysis was carried out in 100 mL autoclave bombs at 90–130 °C for 20–100 min. After acid hydrolysis, an aliquot was withdrawn for xylose analysis.

### Inhibitors adsorption by PS-DVB resin

Hydrophobic PS-DVB resin was used to remove soluble lignin, furfural, and HMF from PHL. Specifically, resin particles were sealed inside of a Chromafex glass column (40 × 2 cm) and then sequentially washed with water, ethanol, and water to remove the preservative salts, adsorbed impurities, and ethanol, respectively. Next, 500 mL of the acid-hydrolyzed PHL (A-PHL) was passed through the resin column by a pump at different flow rates (0.5–4 mL/min). The optimal flow rate was chosen based on the resin’s ability to remove fermentation inhibitors. The other conditions were selected according to the work in Narron et al. [27]. The permeated solution (purified A-PHL) was collected and termed P-A-PHL. After elution of P-A-PHL, 500 mL of deionized water was passed through to collect any soluble sugars that remained unadsorbed within the column. Next, 1.5 L of anhydrous ethanol was eluted through the column to desorb the adsorbates. A final water wash was applied to remove ethanol prior to recovery and reuse of the resin. The efficiency of reused resin was evaluated by adsorbing the fermentation inhibitors in A-PHL for 5 cycle.

### Fermentation of PHL into ethanol

*Pichia stipitis* was used to ferment xylose into ethanol. To activate and inoculate the *P. stipitis*, the yeast was loaded into seed culture solution containing 20 g/L xylose, 3 g/L peptone, and 5 g/L yeast extract and cultured for 72 h at 30 °C under shaking at 150 rpm. Once the density of the yeast reached 15 g/L (cell dry weight), the yeast cells were harvested by centrifugation and then inoculated into the fermentation media, which contained 0.08 g/L MgSO_4_, 2.5 g/L KH_2_PO_4_, 0.25 g/L CaCl_2_, 0.24 g/L urea and P-A-PHL solutions containing 30 g/L and 50 g/L xylose. The pH of fermentation media were controlled at 6.0. Xylose fermentation was carried out at 30 °C with 150 rpm shaking for 36 h. To evaluate the difference in the fermentability difference of A-PHL after purification, A-PHL samples with the same concentrations were also fermented under the same conditions. All fermentations were carried out in a 250-mL Erlenmeyer flask with a working volume of 50 mL. Fermentation was performed with no oxygen supply, but the flasks were not strictly anaerobic due to the use of cotton wool plugs as seals for the flasks [52]. During fermentation, an aliquot was withdrawn for xylose and ethanol analysis at regular intervals on a sterile workbench. Ethanol yield was calculated according to the following equation:

$$ {\text{Ethanol yield (\% )}} = \frac{{\text{Produced ethanol (g)}}}{{{\text{Consumed xylose}}\, \times 0.46{\text{ (g)}}}} \times 100\% . $$

### Whole-cell catalysis of PHL into xylonic acid

*Gluconobacter oxydans* NL71 was used to ferment xylose into XA by whole-cell catalysis. To activate and inoculate *G. oxydans*, the bacterium was loaded into a seed culture solution containing 80 g/L of sorbitol and 8 g/L of yeast extract and cultured at 30 °C under shaking at 220 rpm for 24 h. Once the cell density reached 4 g/L (cell dry weight), cells were harvested by centrifugation and then inoculated into the fermentation media. This fermentation media contained 5 g/L MgSO_4_, 0.5 g/L KH_2_PO_4_, 2 g/L K_2_HPO_4_, 5 g/L (NH_4_)_2_SO_4_, 5 g/L yeast extract and P-A-PHL solutions containing 40 g/L, 90 g/L and 150 g/L xylose. The pH of fermentation medias were controlled at 5.0 To evaluate the fermentability difference of A-PHL after purification, A-PHL with the same concentrations were also fermented at the same conditions. Whole-cell catalysis of A-PHL and P-A-PHL solutions were carried out at 30 °C with 220 rpm shaking for 48 h. All fermentations were carried out in a 250-mL flask with baffles and a working volume of 50 mL. The fermentation flasks were sealed with Parafilm with 0.22 μm pores to allow sterile air into the flask for *G. oxydans* fermentation [53]. During fermentation, an aliquot was withdrawn for xylose and XA analysis at regular intervals on a sterile workbench. XA yield was calculated according to the following equation:

$$ {\text{XA yield (\% )}} = \frac{{\text{Produced XA (g)}}}{{{\text{Consumed xylose}}\, \times 1.1{\text{ (g)}}}} \times 100\% . $$

### Analytical methods

Monosaccharide (glucose and xylose), ethanol, and fermentation inhibitor (furfural and HMF) concentrations were detected by high performance liquid chromatography (HPLC) system consisting of an Aminex HPX-87H column with 50 mM H_2_SO_4_ as the eluent at a flow rate of 0.6 mL/min [54].

The concentration of oligosaccharides in PHL was determined by back calculation using the xylose concentrations before and after acid hydrolysis. Specifically, an aliquot of hydrolyzate was mixed with sulfuric acid to reach a 4% acid concentration and hydrolyzed at 121 °C for 60 min. The concentration of oligosaccharides was calculated from the differences in the respective monosaccharide contents before and after acid hydrolysis, which were also detected using the above HPLC system.

The concentration of XA was determined by a high performance anion-exchange chromatography (HPAEC) system using a CarboPacTM PA10 column and NaOH and sodium acetate as eluents at a flow rate of 0.3 mL/min. The separation method to analyze the XA concentrations in HPAEC was performed according to the work of Zhou and Xu [55].

The concentrations of soluble lignin in PHL, A-PHL, and P-A-PHL were determined by using the Lambert–Beer law (A = Ɛbc), in which the absorbance was obtained from UV spectrophotometry at 280 nm. To avoid lignin overestimation due to furfural and HMF, which also exhibit UV absorption at similar absorption wavelengths as lignin (*λ* = 280 nm), PHL, A-PHL, and P-A-PHL were subjected to borohydride reduction to render UV-silent furfuryl alcohols according to the work of Narron et al. [27] and Chi et al. [56]. The borohydride-treated solutions were used to determine the lignin amount based on UV spectrophotometry.

The chemical structures of PHL, A-PHL, and P-A-PHL were recorded by 2D-HSQC on a Bruker AVIII 600 MHz spectrometer. 50 mg of dried material was dissolved in 0.5 mL DMSO-*d*_6_ to obtain 2D-HSQC spectra according to our previous work [32].

### Statistical analysis

All data were examined in three replications and presented as means ± STD (*n* = 3). Statistical analysis was performed using the one-way analysis of variance (ANOVA) using SPSS statistical software (2008).

## Supplementary information

**Additional file 1: Table S1.** Composition analysis of the A-PHL after being treated by PS-DVB resin column at different flow rate (g/L). **Table S2.** The re-use efficiency of regenerated resin to after different cycle times.

## Data Availability

All data generated and analyzed in this study are included in this published article.

## Abbreviations

PHL: Pre-hydrolysis liquor
PS-DVB: Polystyrene divinylbenzene
A-PHL: Acid-hydrolyzed PHL
P-A-PHL: PS-DVB treated A-PHL
XA: Xylosic acid
HMF: Hydroxymethylfurfural
p-DADMAC: Polydiallyldimethylammonium chloride
CPAM: Cationic polyacrylamide
2D-HSQC: Two-dimensional heteronuclear single-quantum coherence
NMR: Nuclear magnetic resonance
HPLC: High performance liquid chromatography
HPAEC: High performance anion-exchange chromatography
NREL: National Renewable Energy Laboratory
PNNL: Pacific Northwest National Laboratory
