# Supplementary material for: Removal of fermentation inhibitors from pre-hydrolysis liquor using polystyrene divinylbenzene resin
Source: Biotechnol Biofuels. 2020 Nov 12;13:188. doi: 10.1186/s13068-020-01828-3 (PMC7664058; doi:10.1186/s13068-020-01828-3)
Supplement: Supplementary file 1 — Additional file 1: Table S1. Composition analysis of the A-PHL after being treated by PS-DVB resin column at different flow rate (g/L). Table S2. The re-use efficiency of regenerated resin to after different cycle times. [file 13068_2020_1828_MOESM1_ESM.docx]

**Table S1** Composition analysis of the A-PHL after being treated by PS-DVB resin column under different flow rate (g/L)

| Feed flow rate (mL/min) | A-PHL ^a^ | 0.5 | 1 | 2 | 3 | 4 |
| --- | --- | --- | --- | --- | --- | --- |
| Xylose | 101.1±1.1 | 98.2±0.1 | 97.9±0.9 | 97.1±0.9 | 96.9±1.1 | 97.3±1.1 |
| Glucose | 8.8±0.3 | 8.5±1.1 | 8.5±0.3 | 8.4±0.1 | 8.4±0.1 | 8.2±0.3 |
| Arabinose | 4.5±0.2 | 4.4±0.1 | 4.4±0.7 | 4.3±0.5 | 4.3±0.1 | 4.1±0.1 |
| HMF | 1.9±0.1 | 0.04±0.01 | 0.05±0.03 | 0.04±0.01 | 0.12±0.03 | 0.19±0.07 |
| Furfural | 7.8±1.2 | 0.5±0.2 | 0.5±0.1 | 0.6±0.1 | 1.1±0.3 | 2.8±0.4 |
| Soluble lignin | 42.9±0.6 | **1.9**±0.3 | 1.9±0.5 | 2.1±0.3 | 2.9±1.5 | 4.2±0.1 |

^a^ feed stock solution used to purify by PS-DVB resin column

Table S2 The reuse efficiency of regenerated resin after different cycle

|  | A-PHL ^c^  (g/L) | 1 ^st^ time | | 2 ^nd^ time | | 3 ^rd^ time | | 4 ^th^ time | | 5 ^th^ time | |
| --- | --- | --- | --- | --- | --- | --- | --- | --- | --- | --- | --- |
|  |  | Concentration  (g/L) | Efficiency (%) | Concentration  (g/L) | Efficiency (%) | Concentration  (g/L) | Efficiency (%) | Concentration  (g/L) | Efficiency (%) | Concentration  (g/L) | Efficiency (%) |
| Xylose ^a^ | 101.1±1.1 | 97.1±0.9 | 96.1±1.0 | 96.8±1.5 | 95.7±1.7 | 95.6±0.5 | 94.5±0.5 | 95.1±0.5 | 94.1±0.6 | 95.1±0.2 | 94.1±0.2 |
| Glucose ^a^ | 8.8±0.3 | 8.4±0.1 | 95.1±1.4 | 8.4±0.2 | 95.1±2.1 | 8.1±0.2 | 91.7±3.3 | 8.2±0.2 | 92.8±2.3 | 8.1±0.5 | 92.4±7.0 |
| Arabinose ^a^ | 4.5±0.2 | 4.3±0.5 | 94.1±4.6 | 4.3±0.4 | 94.8±10.0 | 4.4±0.3 | 97.8±6.5 | 4.2±0.1 | 93.3±1.8 | 4.3±0.1 | 95.6±3.6 |
| HMF ^b^ | 1.9±0.1 | 0.04±0.01 | 97.9±0.4 | 0.05±0.02 | 97.2±1.2 | 0.06±0.00 | 97.0±0.2 | 0.06±0.03 | 99.2±0.4 | 0.05±0.02 | 99.3±0.3 |
| Furfural ^b^ | 7.8±1.2 | 0.6±0.1 | 91.9±1.6 | 0.7±0.1 | 91.5±1.6 | 0.6±0.1 | 92.3±2.1 | 0.6±0.1 | 92.7±1.6 | 0.8±0.2 | 90.2±3.7 |
| Soluble lignin ^b^ | 42.9±0.6 | 2.1±0.3 | 95.1±0.8 | 2.1±0.1 | 95.0±0.4 | 2.2±0.3 | 94.8±0.8 | 2.5±0.3 | 94.2±0.8 | 2.4±0.2 | 94.3±0.7 |

^a^ Efficiency of recovery yield of carbohydrate

^b^ Efficiency of remove yield of lignin and sugar by-products

^c^ The original stock for re-use experiments
